# Rewriting CAR-T cell fate: CRISPR/Cas gene editing for solid tumor therapy

**DOI:** 10.3389/fimmu.2026.1910092

**Published:** 2026-07-28

**Authors:** Wenjing Liu, Jiayi Gu, Chenghao Xie, Bing Du, Mingyao Liu, Jiqin Zhang

**Affiliations:** Shanghai Frontiers Science Center of Genome Editing and Cell Therapy, Shanghai Key Laboratory of Regulatory Biology, Institute of Biomedical Sciences and School of Life Sciences, East China Normal University, Shanghai, China

**Keywords:** CAR-T, CRISPR/Cas, gene editing, solid tumors, tumor immunotherapy

## Abstract

Although chimeric antigen receptor T (CAR-T) cell therapy has achieved remarkable success in hematological malignancies, its therapeutic efficacy in solid tumors remains limited by several challenges, including insufficient tumor infiltration, T cell exhaustion and the immunosuppressive tumor microenvironment (TME). CRISPR/Cas, a third-generation gene editing technology developed in recent years, is characterized by its simplicity and high efficiency. This technology has demonstrated broad application potential across multiple fields and has emerged as a powerful tool for improving CAR-T cell therapy. In this review, we summarize recent advances in the application of CRISPR/Cas gene editing technology to enhance the antitumor activity of CAR-T cells against solid tumors. We also discuss the key challenges currently faced and systematically propose potential strategies for overcoming the limitations.

## Introduction

Tumor immunotherapy has advanced rapidly in recent years, among which chimeric antigen receptor T (CAR-T) cell therapy holds great promise (1–3). CAR-T cell therapy relies on genetic engineering to modify T cells, so that they stably express functional chimeric antigen receptors (CARs) (4). A typical CAR consists of an extracellular antigen-binding domain, a transmembrane domain, and intracellular signaling domains. The extracellular domain specifically recognizes tumor-associated antigens and initiates immunological synapse formation, whereas the intracellular domains integrate costimulatory signals, such as those derived from CD28 or 4-1BB, together with the T cell activation signal mediated by CD3ζ (5–7). This dual-signaling design ensures efficient activation, expansion, and cytotoxic activity of CAR-T cells against target cells (8). In addition, CAR-T cells can induce apoptosis of target cells through the Fas/FasL pathway or secrete cytokines such as interferon-γ (IFN-γ) and tumor necrosis factor-α (TNF-α), thereby exerting indirect antitumor effects (9). In the treatment of hematologic malignancies, CAR-T immunotherapy has achieved unprecedented breakthroughs (10–12). To date, several CAR-T cell products, including axicabtagene ciloleucel and tisagenlecleucel, have been approved, marking the successful clinical translation and commercialization of this therapeutic modality (13, 14). Clinical studies have demonstrated that CAR-T cell therapy can induce deep and durable remissions in patients with relapsed or refractory acute lymphoblastic leukemia (ALL), B-cell non-Hodgkin lymphoma (B-NHL), and multiple myeloma (MM) (10–14). Moreover, beyond tumor treatment, CAR-T cell therapy has also shown immense potential in treating autoimmune diseases by selectively eliminating or modulating pathologically activated immune cell populations (15, 16). Several recent clinical studies have shown that CAR-T cell therapy can achieve profound disease remission in systemic lupus erythematosus and other refractory autoimmune diseases (15, 17).

An increasing number of studies have shown that the activation, persistence, and resistance to immunosuppression of T cells are regulated by multiple intracellular programs, including transcriptional, epigenetic, and metabolic pathways (18–20). Emerging evidence demonstrates that transcriptional programs directly dictate T cell function. For example, during prolonged antitumor responses, antigen-specific T cells gradually lose their proliferative capacity and enter an exhausted state, which is closely associated with the upregulation of exhaustion-related transcription factors such as TOX and NR4A (21–23), as well as downregulation of memory-associated ones including TCF7 and KLF2 (24–26). In addition, epigenetic mechanisms, including DNA methylation, histone modification, and chromatin remodeling, dynamically regulate the status of T cells (27–29). For instance, aberrant histone deacetylase (HDAC) activity may impair the persistence of cytotoxic T cells through epigenetic silencing of stemness-related genes (30, 31), whereas *de novo* methyltransferases repress genes with memory functions by regulating DNA methylation levels (32, 33). Furthermore, metabolic regulation has been demonstrated to be essential for T cell function: memory T cells primarily rely on oxidative phosphorylation, whereas effector T cells depend more on glycolysis (34). Thus, modulating these intracellular pathways represents a promising approach to restore CAR-T cell fitness and improve the antitumor efficacy. In this context, the CRISPR/Cas system, a gene editing platform derived from the adaptive immune system of bacteria, has emerged as a powerful tool to rewire CAR-T cells (35–37). Guided by RNA molecules, Cas nucleases can target specific DNA sequences to enable gene knock-out and knock-in, base editing, and transcriptional regulation (35). Owing to its simplicity, high efficiency, and precise targeting capability, CRISPR/Cas provides a versatile strategy for overcoming the current limitations of CAR-T cell therapy (35, 36).

## Challenges in CAR-T cell therapy against solid tumors

Although CAR-T cell therapy has achieved remarkable success in hematologic malignancies, its application in solid tumors, which account for more than 90% of all cancers, remains highly challenging (38, 39). Unlike hematologic malignancies, solid tumors are characterized by insufficient vascularization and a dense extracellular matrix (ECM)-rich TME, which together form physical barriers to CAR-T cell trafficking and infiltration (38, 40, 41). As a result, CAR-T cells often fail to efficiently home from the circulation to the tumor sites. Even when they reach the tumor periphery, they may be unable to penetrate the stromal barrier and enter the tumor parenchyma, thereby limiting their direct contact with tumor cells and ultimately impairing the cytotoxic activity (40, 41).

The functional impairment of CAR-T cells is further exacerbated by the complex immunosuppressive network within the TME (40, 42). Solid tumors are enriched with suppressive cellular components, including myeloid-derived suppressor cells (MDSCs), regulatory T cells (Tregs), and tumor-associated macrophages (TAMs) (43–45). These cells inhibit CAR-T cell activation and proliferation, either through direct cell-cell interactions or by secreting immunosuppressive cytokines such as transforming growth factor-β (TGF-β) and interleukin-10 (IL-10) (43, 45–47). In parallel, metabolic stress within the TME, including hypoxia, the accumulation of acidic metabolites, and the depletion of essential nutrients such as glucose, disrupts the metabolic homeostasis of CAR-T cells and suppresses their effector functions (48).

Persistent antigen stimulation within the TME is another major factor limiting the therapeutic efficacy of CAR-T cells (49). Continuous engagement of CAR-T cells with highly expressed target antigens on tumor cells triggers chronic signaling activation, which in turn induces the upregulation of immune inhibitory receptors such as PD-1 and LAG-3 (50, 51). In this process, CAR-T cells progressively enter a functionally exhausted state, accompanied by a reduction in memory T cell subsets, and ultimately lose their capacity to mediate sustained antitumor activity (49–51).

Another major challenge is the heterogeneity of antigen expression in solid tumors (52). Some tumor cell subpopulations may lose the expression of target antigen, allowing them to evade CAR-T cell recognition. Consequently, CAR-T cells fail to eliminate all of tumor cells effectively, thereby facilitating tumor antigen escape. Furthermore, some tumor target antigens may also be expressed at similar levels in certain normal tissues (53, 54). Under such circumstances, CAR-T cells may attack healthy tissues while killing tumor cells, leading to on-target and off-tumor toxicity (55). Collectively, these reasons make it difficult for CAR-T cells to achieve durable and effective antitumor responses in solid tumors, thus highlighting the need for optimization of CAR-T cells through gene editing technologies such as the CRISPR/Cas system.

## Overview of gene editing technologies

Since its emergence in the 1980s, gene editing technology has gradually become one of the indispensable tools in the field of life sciences and medicine (56). Major breakthroughs were achieved between the 1990s to the 2010s with the development of zinc-finger nucleases (ZFNs) and transcription activator-like effector nucleases (TALENs) (57, 58). ZFNs enable specific recognition and cleavage of defined DNA sequences through engineered zinc-finger protein-nuclease fusion constructs, thereby making precise gene editing possible (58). TALENs are generated by fusing modular TALE proteins with the *Fok*I endonuclease, enabling precise recognition and cleavage of specific DNA sequences (59). Compared with ZFNs, TALENs offer several advantages, including simpler design, higher specificity and lower off-target effects (59). However, both ZFNs and TALENs are still limited by complex design procedures, long development cycles, and substantial technical barriers in target customization. These limitations have greatly restricted their broad applications in both basic research and clinical translation (59, 60). In 2012, the discovery and development of the CRISPR/Cas system revolutionized the field of gene editing (61, 62). Due to its high efficiency, simplicity, precise targeting capability, and potential for multiplex editing, CRISPR/Cas tool has rapidly become mainstream gene editing methods. It is now widely used in gene function screening, gene and cell therapy, molecular diagnostics, genetic breeding, and bioreactor engineering (63). Based on the CRISPR/Cas platform, researchers have further developed a series of next-generation gene editing technologies. Among them, base editing (BE) enables precise single-nucleotide substitutions without introducing DNA double-strand breaks (DSBs), thereby significantly reducing the risk of genotoxic damage (64). This approach has become an ideal strategy for correcting point mutations associated with hereditary diseases, such as sickle cell disease and certain inherited metabolic disorders (65). In 2019, prime editing (PE) was introduced as a more versatile gene editing tool. PE can mediate targeted substitutions, insertions, and deletions without relying on DSBs, offering improved controllability and broader gene editing scope (66). It is particularly suitable for repairing disease-causing point mutations as well as insertions and deletions (67–69). In parallel, other strategies for large-fragment genomic integration are also being rapidly developed, providing new powerful technologies. The iterative advancement of these gene editing technologies has greatly expanded the toolbox available for CAR-T cell engineering and promoted the development of next-generation CAR-T cell therapies.

## Reprogramming CAR-T cells by CRISPR/Cas gene editing

### Inhibitory receptors

**Figure 1:**
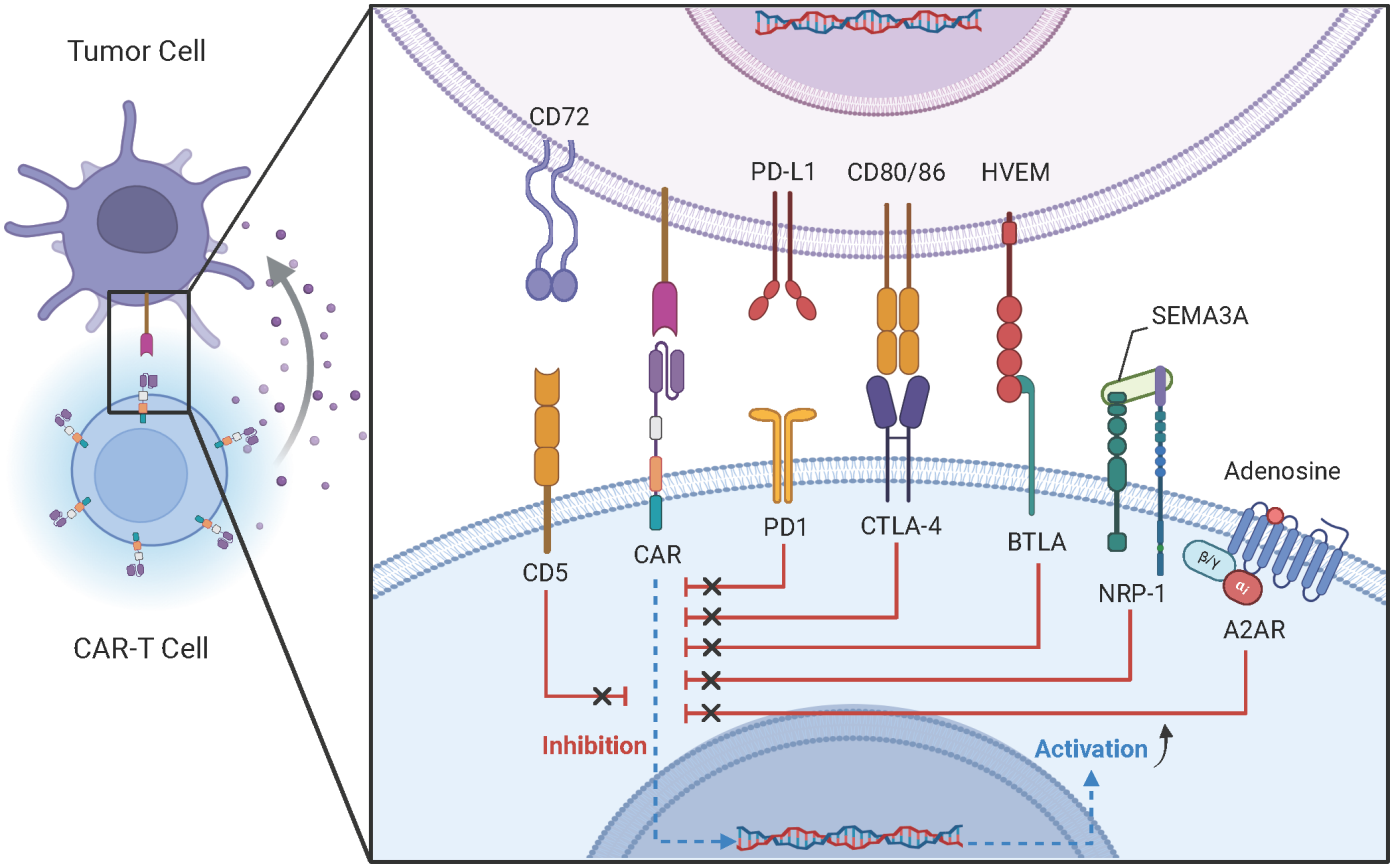
Reprogramming CAR-T cells via CRISPR/Cas gene editing of inhibitory receptors for solid tumor therapy. CAR-T cell therapy faces multiple challenges in the treatment of solid tumors. CRISPR/Cas-mediated gene editing of inhibitory receptors (e.g., PD1, CTLA-4, BTLA, A2AR, NRP-1 and CD5) represents a promising strategy to enhance CAR-T cell function against solid tumors. Created with BioRender.com.

Inhibitory receptors (IRs) are a class of transmembrane glycoproteins expressed on the surface of T cells and belong to the immunoglobulin superfamily (IgSF). By interacting with their corresponding ligands, these receptors transmit inhibitory signals that limit CAR-T cell function, promote exhaustion, and weaken anti-tumor responses. Within the TME of solid tumors, tumor cells, stromal cells, and immunosuppressive immune cells frequently express high levels of these ligands, resulting in persistent exposure of CAR-T cells to inhibitory signaling. This process largely contributes to the limited efficacy of CAR-T cell therapy in solid tumors. Therefore, genetic modulation of IRs has emerged as a promising strategy for enhancing CAR-T cell function and persistence (**Figure 1** and **Table 1**).

**Table 1:** Overview of gene editing targets for CAR-T cell engineering against solid tumors.

Gene	Protein	Molecular category	Editing outcome	Tumor model	Reference
PDCD1	PD-1	Inhibitory receptor	Enhanced tumor control and persistence	HCC Breast cancer	Guo et al. (70) and Hu et al. (71)
CTLA-4	CTLA-4	Inhibitory receptor	Enhanced antitumor cytotoxicity	Colon cancer	Shi et al. (75)
BTLA	BTLA	Inhibitory receptor	Enhanced antitumor activity	Pancreatic cancer Prostate cancer Melanoma	Guruprasad et al. (76)
NRP-1	NRP-1	Inhibitory receptor	Enhanced tumor infiltration and persistence	Melanoma	Liu et al. (79)
A2AR	A2AR	Inhibitory receptor	Improved tumor control and survival	Breast cancer	Giuffrida et al. (80)
CD5	CD5	Inhibitory receptor	Improved survival and persistence	Pancreatic cancer Melanoma	Patel et al. (81)
BATF	BATF	Transcription factor	Enhanced tumor clearance and persistence	Pancreatic cancer NSCLC	Zhang et al. (87)
TOX	TOX	Transcription factor	Reduced exhaustion and enhanced persistence	Melanoma	Seo et al. (88)
NR4A1/2/3	NR4A1/2/3	Transcription factor	Reduced exhaustion and enhanced tumor control	NSCLC Melanoma	Lam et al. (89), Srirat et al. (90), and Nakagawara et al. (91)
EGR2	EGR2	Transcription factor	Improved expansion and tumor suppression	Pancreatic cancer Prostate cancer	Jung et al. (92)
PRDM1	BLIMP-1	Transcription factor	Enhanced antitumor activity and persistence	Melanoma	Yoshikawa et al. (93)
ITK	ITK	Kinase	Enhanced tumor clearance and persistence	Subcutaneous CLL	Fu et al. (95)
MAP4K1	HPK1	Kinase	Reduced exhaustion and enhanced tumor control	Melanoma Breast cancer	Si et al. (97)
DGKA/Z	DGKα/ζ	Kinase	Enhanced antitumor cytotoxicity	Ovarian cancer	Evtimov et al. (99)
ZC3H12A	Regnase-1	Metabolic regulator	Enhanced antitumor activity and persistence	Melanoma Pancreatic cancer NSCLC	Wei et al. (102) and Mai et al. (105)
RC3H1	Roquin-1	Metabolic regulator	Enhanced antitumor cytotoxicity and persistence	Pancreatic cancer NSCLC	Mai et al. (105)
ACAT1	ACAT1	Metabolic regulator	Improved expansion and antitumor cytotoxicity	Melanoma	Yang et al. (109)
TET2	TET2	Epigenetic regulator	Reduced exhaustion and enhanced expansion	Osteosarcoma Prostate cancer	Dimitri et al. (112) and Jain et al. (113)
DNMT3A	DNMT3A	Epigenetic regulator	Reduced exhaustion and enhanced antitumor activity	Glioblastoma Osteosarcoma	Prinzing et al. (115)
SUV39H1	SUV39H1	Epigenetic regulators	Enhanced expansion and persistence	Prostate cancer NSCLC	Jain et al. (119) and López-Cobo et al. (120)

HCC, hepatocellular carcinoma; NSCLC, non-small cell lung cancer; CLL, chronic lymphocytic leukemia.

Among identified IRs, PD-1 is a well-known immune checkpoint molecule, which play important roles in suppressing T cell functions. PD-1 is markedly upregulated following T cell activation, and disruption of PD-1 enables CAR-T cells to resist PD-L1-mediated inhibition within tumors, thereby improving *in vivo* expansion and tumor control (70, 71). This strategy has also been evaluated in several clinical studies. A clinical study in patients with non-small cell lung cancer (NSCLC) showed that, among those treated with *PDCD1-*knockout anti-MUC1 CAR-T cells, 11 patients achieved stable disease (SD), while 9 patients experienced progressive disease (PD). Symptom improvement and *in vivo* expansion of CAR-T cells were observed after treatment (72). Another clinical study in patients with mesothelin-positive solid tumors showed that, following infusion of *PDCD1*- and *TRAC*-knockout mesothelin-specific CAR-T cells, 2 of 15 patients achieved SD, while 7 patients achieved SD at 3–4 weeks after treatment (73). CTLA-4 and BTLA are also well-characterized IRs that play important roles in suppressing CAR-T cell function. In CAR-T cells containing a CD28 costimulatory domain, high CTLA-4 expression is closely associated with T cell dysfunction (74). Using CRISPR/Cas-mediated gene editing, Shi et al. demonstrated that *CTLA-4* knockout significantly enhanced the cytotoxic activity of T cells against colon cancer cells both *in vitro* and *in vivo* (75). Recently, the BTLA-HVEM signaling axis has been identified as a critical pathway restricting T cell efficacy. Disruption of BTLA markedly enhanced the antitumor activity of CAR-T cells *in vivo* without obvious toxicity (76). Beyond classical immune checkpoints, several additional IRs have attracted attention in recent years. Neuropilin-1 (NRP-1) was shown to maintain the immunosuppressive function of tumor-associated Tregs (77), and its blockade improves anti-tumor immunity and promotes the development of memory-like T cell phenotypes (78). Liu et al. demonstrated that CRISPR-mediated deletion of NRP-1 could enhance tumor infiltration and long-term antitumor activity of T cells (79). The adenosine A2A receptor (A2AR), which is highly expressed in T cells, mediates adenosine-induced immunosuppression in the TME. CRISPR/Cas-mediated knockout of *A2AR* has been reported to significantly improve tumor control by CAR-T cells, accompanied by increased secretion of effector cytokines such as IFN-γ and TNF-α, and reduced sensitivity to adenosine-induced immunosuppression (80). In an orthotopic breast cancer model, A2AR-deficient CAR-T cells significantly prolonged overall survival, achieving an 80% survival rate at the study endpoint (day 60 post-infusion), compared with 0% in the control group. Notably, mice that had been previously cured with *A2AR-*knockout CAR-T cells completely rejected a subsequent tumor rechallenge, indicating the establishment of durable immunological memory. Additionally, genetic disruption of *CD5*, a negative regulator of T cell activation, lowers the activation threshold of CAR signaling and enhances the anti-tumor activity of CAR-T cells in a subcutaneous pancreatic cancer model (81). This enhancement substantially prolonged the median overall survival from 46 to 102 days compared with control CAR-T cells.

Due to the complexity and redundancy of IR regulatory networks, targeting a single IR is often insufficient to fully restore CAR-T cell function, as parallel inhibitory pathways can compensate and continue to suppress T cell activity. To overcome this limitation, multiplex gene editing strategies that simultaneously disrupt several IRs, such as PD-1, LAG-3, TIM-3 and CTLA-4, have been proved to be more effective. These combinatorial approaches have shown synergistic effects in multiple preclinical studies by significantly enhancing T cell infiltration, persistence, and cytotoxic activity against solid tumors (82–84).

### Transcription factors

**Figure 2:**
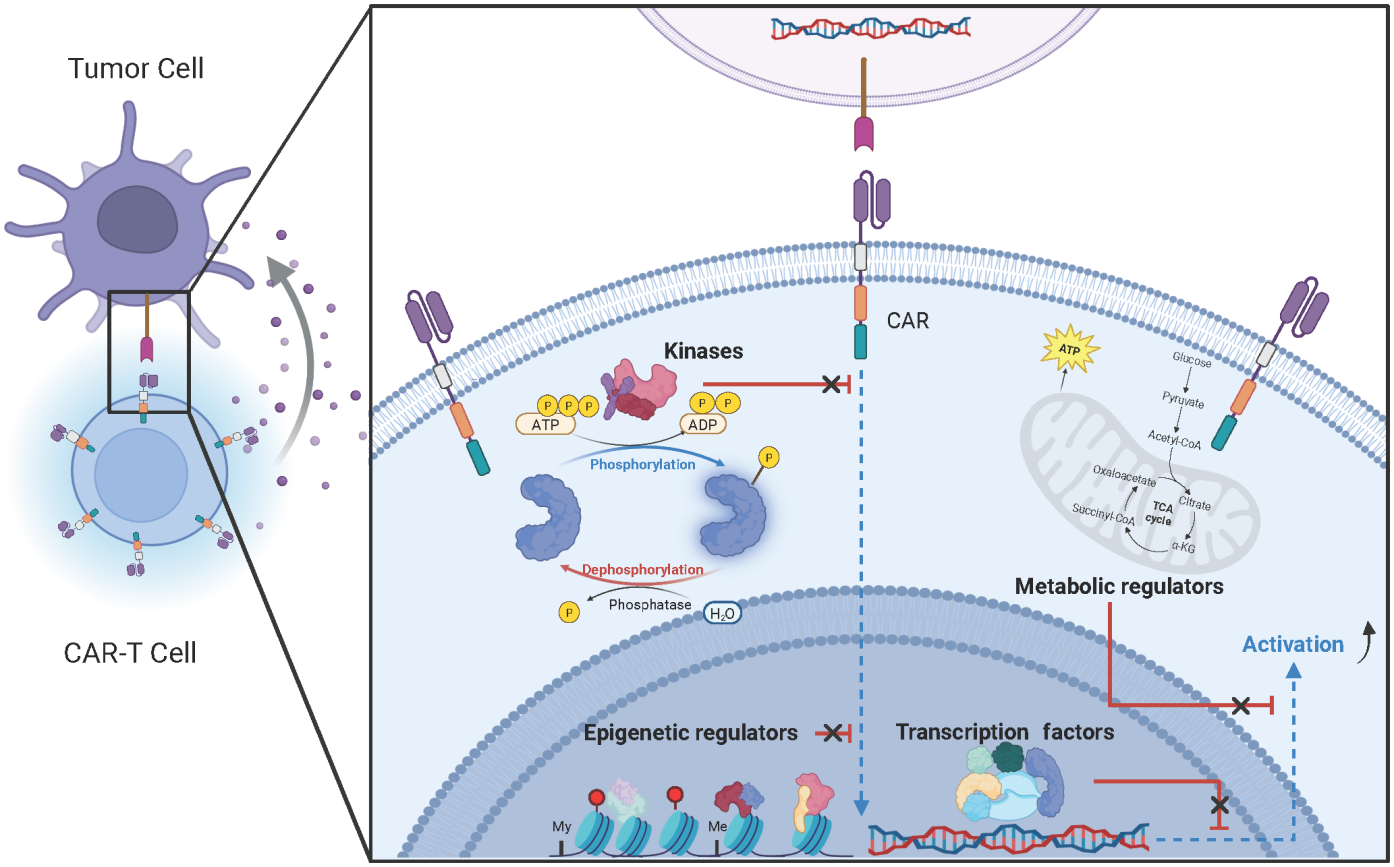
Rewiring CAR-T cells via CRISPR/Cas gene editing of key intracellular factors for solid tumor therapy. The efficacy of CAR-T cell therapy against solid tumors remains constrained by multiple challenges. CRISPR/Cas gene editing holds great promise for reprogramming key intracellular factors (including kinases, transcription factors, metabolic and epigenetic regulators) in CAR-T cells to overcome these limitations. Created with BioRender.com.

Transcription factors are critical regulatory elements that control T cell fate determination, proliferation and immune function. In the TME of solid tumors, prolonged antigen stimulation, inflammatory signaling, and metabolic stress can dynamically reshape transcriptional regulatory networks, thereby influencing T cell differentiation programs and functional states. Therefore, altering key transcription factors using CRISPR/Cas gene editing technology to reprogram CAR-T cells has emerged as an important strategy to improve therapeutic efficacy in solid tumors (**Figure 2** and **Table 1**).

BATF, a member of the AP-1 transcription factor family, is upregulated upon antigen stimulation. It then forms a transcriptional regulatory complex with IRF4 that cooperatively binds to AP-1-IRF composite elements (AICEs), enabling it to regulate multiple target genes and thereby influence T cell function (85, 86). Zhang et al. reported that deletion of BATF renders CAR-T cells more resistant to exhaustion and enhances their cytotoxic activity in solid tumor models (87). In a pancreatic cancer patient-derived xenograft (PDX) model, intratumoral administration of BATF-deficient CAR-T cells completely eradicated tumors in 80% of the mice. Notably, this effect was observed exclusively under exhaustion-inducing conditions. In contrast, under non-exhausting conditions, overexpression of BATF increased anti-tumor cytotoxicity in a subset of cases. These findings highlight the context-dependent roles of key regulatory molecules and underscore the need for sophisticated gene editing strategies. In addition, the authors found that *IRF4* knockout in CAR-T cells also promoted effector cytokine release and tumor lysis, albeit to a lesser extent than BATF deficiency (87).

TOX has been reported to play central roles in driving T cell exhaustion. Khan et al. demonstrated that TOX can suppress sustained expression of genes associated with effector T cell (T_eff_) differentiation by remodeling chromatin accessibility (23). Subsequent studies further confirmed that TOX partial impairment significantly enhanced the proliferative capacity and cytotoxic activity of CAR-T cells in melanoma models, while maintaining functional persistence even under high tumor burden (88).

The NR4A family (NR4A1, NR4A2, and NR4A3) represents another crucial regulatory axis controlling T cell exhaustion. Chen et al. reported that elevated NR4A expression is closely associated with exhaustion phenotypes (22). Using CRISPR/Cas-edited gene editing, Lam et al. individually knocked out *NR4A1*, *NR4A2*, or *NR4A3* and found that *NR4A3* knockout alone significantly enhanced the cytokine production and cytotoxicity in anti-ROR1 CAR-T cells (89). Moreover, the combination of *NR4A3* knockout with c-Jun overexpression produced a synergistic enhancement of CAR-T cell antitumor activity in a lung cancer xenograft model (89). Deletion of both NR4A1 and NR4A2 has also been shown to promote the accumulation of TCF1^+^ stem-like progenitor exhausted T (Tpex) cells within the TME (90). Consistently, a further study demonstrated that a *NR4A1/2/3* triple knockout strategy enabled CAR-T cells to resist exhaustion while retaining memory characteristics, leading to robust antitumor responses *in vivo* (91). In a subcutaneous A549 lung adenocarcinoma model, this approach induced rapid tumor regression as early as day 7 post-infusion, with over 50% of treated mice maintaining durable tumor control through day 40.

In other studies, Jung et al. showed that knockout of *EGR2* in CAR-T cells attenuated IFN-I driven dysfunction, improved expansion and cytotoxic capacity, and increased the proportion of early memory-like T cells (92). In a subcutaneous prostate cancer xenograft model, CAR-T cells lacking EGR2 significantly suppressed tumor growth, with all eight treated mice showing effective tumor control by day 60 post-infusion. *PRDM1*, which encodes the transcription factor BLIMP-1, is a key regulator of terminal differentiation. Persistent PRDM1 expression accelerates the transition of T cells from an effector state toward exhaustion. Yoshikawa et al. demonstrated that *PRDM1* knockout not only preserved early memory characteristics of T cells but also restored certain memory-like features in terminally differentiated T cells, thereby enhancing the proliferative potential and long-term persistence of CAR-T cells (93). In a subcutaneous A375 melanoma model, both wild-type and BLIMP-1-deficient CAR-T cells effectively eliminated primary tumors. However, upon tumor rechallenge, CAR-T cells lacking BLIMP-1 exhibited significantly enhanced tumor control and superior *in vivo* persistence through day 24, whereas mice receiving wild-type CAR-T cells showed rapid tumor progression.

### Kinases

Kinases function as molecular switches within cellular signaling networks and play critical roles in regulating T cell activity. Among them, negative regulatory kinases can suppress T cell receptor (TCR) and CAR signaling, attenuate cytokine responses, and accelerate exhaustion, thereby acting as one of major bottlenecks that limit the efficacy of CAR-T cells in solid tumors. Accordingly, CRISPR/Cas-mediated disruption of these inhibitory kinases has emerged as an effective strategy for alleviating signaling constraints and enhancing the antitumor efficacy of CAR-T cells (**Figure 2**, **Table 1**).

IL-2-inducible T cell kinase (ITK) is a critical downstream kinase in the TCR signaling pathway (94). Under chronic antigen stimulation, sustained ITK activation can upregulate exhaustion-associated transcription factors, accelerate the differentiation of CAR-T cells toward a terminally exhausted phenotype, and promote cell apoptotic signaling. As reported by Zheng Fu et al., the knockout of *ITK* not only reduced the expression of exhaustion markers but also increased the levels of memory-associated transcription factors, thus enhancing the sustained cytotoxic activity of CAR-T cells (95). In a subcutaneous xenograft model of the chronic lymphocytic leukemia (CLL) cell line MEC1, *ITK*-knockout CAR-T cells achieved complete tumor regression at day 14 post-infusion and exhibited approximately two-fold higher expansion than control CAR-T cells.

Hematopoietic progenitor kinase 1 (HPK1) is a serine/threonine kinase encoded by *MAP4K1* that acts as a negative regulator of T cell signaling. It exerts this function by phosphorylating SLP-76 and GADS, thereby recruiting the inhibitory adaptor protein 14-3-3. This process promotes dissociation of the SLP-76-GADS complex from LAT, leading to disassembly of TCR signaling microclusters and ultimately delivering a “stop” signal that fine-tunes the magnitude of T-cell immune responses (96). Under prolonged tumor-antigen stimulation, HPK1 can also promote JNK signaling and increase TOX expression, driving the upregulation of exhaustion markers such as PD-1 and LAG-3. CRISPR/Cas-mediated deletion of HPK1 in CAR-T cells has been reported to enhance the secretion of effector molecules including Granzyme B and TNF-α and reduce the degree of T cell exhaustion, thus augmenting tumor clearance (97). In a subcutaneous SK-BR-3 breast cancer xenograft model, HPK1-deficient anti-HER2 CAR-T cells markedly inhibited tumor growth and improved the survival of treated mice to 50% by day 60 post-infusion, compared with 0% survival in the wild-type CAR-T cell group.

Diacylglycerol kinases (DGKs) belong to an important kinase family involved in the metabolism of lipid second messengers (98). In the TME, suppressive signals can enhance DGK activity, leading to excessive consumption of diacylglycerol (DAG), and impairing the activation of downstream transcription factors such as NF-κB and AP-1, thereby restricting CAR-T cell activation. The dual knockout of *DGKA* and *DGKZ* in CAR-T cells has been shown to restore DAG signaling, consequently increasing cytokine secretion, improving the recruitment of other immune cell populations and enhancing cytotoxicity against ovarian cancer cells (99). In a subcutaneous OVCAR-3 ovarian cancer xenograft model, CAR-T cells lacking either DGKα or DGKζ suppressed tumor growth more effectively than control CAR-T cells, although tumors relapsed around days 40–50 post-infusion. In contrast, dual *DGKA* and *DGKZ* knockout CAR-T cells induced sustained tumor eradication, with 100% survival over the 100-day observation period.

### Metabolic regulators

Factors that affect metabolism serve as critical regulators of intracellular homeostasis and T cell function. They not only influence the survival and activity of T cells by controlling fundamental processes such as energy metabolism, lipid synthesis, and redox balance, but also directly participate in the regulation of T cell activation, proliferation, and differentiation through extensive crosstalk with signaling pathways and transcriptional programs. Thus, reshaping the metabolic state of CAR-T cells via CRISPR/Cas-mediated gene editing has become an attractive strategy to improve their metabolic adaptability, survival, and anti-tumor efficacy within the solid tumor microenvironment (**Figure 2**, **Table 1**).

Regnase-1 is a multifunctional RNA-binding protein that plays a critical role in regulating T cell metabolism and function (100, 101). It maintains immune homeostasis by degrading the mRNAs encoding pro-inflammatory cytokines, such as IL-6 and IL-12, thereby limiting excessive cytokine-mediated immune activation. It also negatively regulates T cell function by degrading effector-associated mRNAs (e.g., *IFNG*, *GZMB*), thereby limiting T cell activation, proliferation, and antitumor activity. Conversely, Regnase-1 inhibition promotes the generation of long-lived effector T cells and enhances antitumor function. Consistently, interfering with Regnase-1 function in T cells was observed to significantly increase the levels of effector cytokines, reduce the expression of exhaustion markers, and enhance the infiltration of endogenous immune cells (102).

Roquin-1, encoded by the *RC3H1* gene, is an RNA-binding protein that plays an important role in the immune system. It primarily functions as a post-transcriptional repressor to inhibit excessive T cell responses and inflammation by promoting the degradation of specific immunomodulatory mRNAs such as *ICOS*, *TNF*, *NFKBIZ* and *IRF4*. It is typically upregulated in solid tumor microenvironments enriched in TGF-β. Roquin-1 can bind to the 3′ untranslated region (3′ UTR) of *ICOS* mRNA, promoting its degradation or translational repression, thereby negatively regulating *ICOS* expression and suppressing T cell activation and effector responses, ultimately maintaining immune homeostasis and preventing excessive immune reactions (103, 104). It has been proved that in a subcutaneous AsPC1 pancreatic cancer xenograft model, *RC3H1*-knockout CAR-T cells increased the survival rate from 0% to 80% by day 80 post-infusion compared with control cells (105). Notably, Regnase-1 and Roquin-1 have been found to interact to form an RNA regulatory complex, cooperatively promoting the degradation of inflammatory mRNAs (106). In line with this, a dual knockout strategy was demonstrated to rejuvenate T cells more significantly than either single knockout alone, by enhancing exhaustion resistance, tumor infiltration and antitumor potency (105).

ACAT1 is a well-known enzyme in lipid metabolism that catalyzes the esterification and storage of free cholesterol. By reducing the level of free cholesterol in the T cell membrane, ACAT1 suppresses TCR signal amplification and impairs immunological synapse maturation, thereby weakening T cell activation, proliferation and tumor-killing capacity (107–109). Therefore, some researchers proposed an ACAT1-targeting strategy to improve CAR-T cell antitumor activity by enhancing cholesterol-dependent metabolic remodeling (109). In a subcutaneous B16F10 melanoma xenograft model, mice treated with ACAT1-depleted CAR-T cells exhibited prolonged survival compared with control CAR-T cells, with survival rate increasing from 0% to 50% by day 30 post-infusion.

### Epigenetic regulators

As key modulators, epigenetic regulators shape gene expression networks and dynamically control the activation and differentiation of T cells through mechanisms such as DNA methylation, histone modification, and chromatin remodeling. In the solid tumor microenvironment, aberrant epigenetic modifications of CAR-T cells often drive T cell exhaustion, impair tumor infiltration, and promote an immunosuppressive status, thereby becoming one of determinants of limited therapeutic efficacy. Disruption of critical epigenetic regulators through CRISPR/Cas-mediated gene editing can reverse inhibitory epigenetic states and enhance the antitumor function of CAR-T cells, thus providing an encouraging strategy for overcoming current barriers in solid tumor treatment (**Figure 2** and **Table 1**).

TET2 is an essential enzyme involved in DNA demethylation. It oxidizes the methyl group of 5-methylcytosine (5mC) to generate 5-hydroxymethylcytosine (5hmC), 5-formylcytosine (5fC) and 5-carboxylcytosine (5caC), thereby playing a critical role in transcriptional silencing and genome stability (110). During lymphocytic choriomeningitis virus (LCMV) infection, TET2 drives the differentiation of stem-like Tpex cells into effector-like terminally exhausted T (Tterm) cells, suggesting that the targeted disruption of *TET2* could possibly enhance T cell activity by promoting functional persistence. Notably, in a clinical study on CLL, TET2 attenuation was found to enhance the memory phenotype of CAR-T cells and in turn lead to a clonal expansion of CAR-T cells in one patient, ultimately achieving a long-term complete remission (111). Further investigation revealed that under chronic antigen stimulation, TET2 deficiency in CAR-T cells restricts their differentiation into Tterm cells, maintains or enriches cell populations with stem/progenitor cell characteristics, and enhances the proliferative capacity and metabolic fitness, which eventually improves the antitumor efficacy (111, 112). However, another study indicated that biallelic *TET2* knockout relieves the inhibition of the BATF3-MYC axis, resulting in antigen-independent uncontrolled proliferation and tissue infiltration of CAR-T cells, which in turn causes severe toxicity (113). To address this issue, it is necessary to incorporate a safety switch to mitigate the risk of uncontrolled proliferation of *TET2*-null CAR-T cells, or to retain attenuated TET2 activity rather than achieving complete enzymatic ablation. For example, Dimitri et al. generated *TET2*-knockout CAR-T cells co-expressing truncated EGFR, which enables rapid elimination via cetuximab-mediated antibody-dependent cell-mediated cytotoxicity (ADCC) should severe toxicity arise (112).

DNMT3A is a well-known *de novo* DNA methyltransferase (114). Under chronic antigen stimulation, elevated DNMT3A expression promotes the differentiation of T cells toward a terminally exhausted phenotype by methylating and silencing effector genes such as *IFNG* and *GZMB*, as well as memory-associated genes including *IL7R* and *BCL6* (114). One study has shown that depletion of *DNMT3A* enables CAR-T cells to maintain a high proliferative potential and more significantly suppress tumor progression in solid tumors (115). In an intraperitoneal osteosarcoma xenograft model, DNMT3A-depleted CAR-T cells exhibited sustained antitumor activity, achieving a 60% survival rate by day 150 post-infusion, compared with 0% in the control group. DNMT1 is a maintenance DNA methyltransferase, which methylates newly synthesized unmethylated nascent DNA strands during DNA replication, thereby ensuring the maintenance and stability of genomic DNA methylation. It was observed that inhibiting DNMT1 can reprogram CAR-T cells into NK-like cells with enhanced antitumor potency (116).

SUV39H1 is a histone methyltransferase that specifically catalyzes H3K9 trimethylation (H3K9me3), which is known to repress gene transcription by altering chromatin structure and interfering with transcription factor binding. On the one hand, during the differentiation of CD8^+^ T cells into effector cells, SUV39H1 catalyzes H3K9me3 around stemness-related genes, thereby suppressing memory-like properties. On the other hand, H3K9me3 modification is also enriched at the promoter regions of genes encoding cytotoxic effector molecules such as GZMB, PRF1 and IFNG, keeping T cells in a repressed state (117, 118). In CAR-T cells, Jain et al. found that knockout of *SUV39H1* reduced the expression of IRs, increased the expression of memory-associated transcription factors, and accelerated cell expansion, accordingly promoting tumor rejection after repeated stimulation (119). In a metastatic prostate cancer model, SUV39H1-deficient CAR-T cells significantly prolonged survival compared with control CAR-T cells, achieving a 60% survival rate by day 80 post-infusion versus 0% in controls. Another study also suggested that the loss of SUV39H1 improves the durability of CAR-T cells by relieving epigenetic silencing of memory- and effector-associated genes, which induces the formation of T memory stem cells (Tscm) (120). In an intravenous A549 lung cancer xenograft model, SUV39H1-depleted CAR-T cells markedly prolonged survival compared with wild-type CAR-T cells, with survival rate increasing from 29% to 86% by day 188 post-infusion.

## Challenges and future perspectives

### Expanding gene editing technologies

Although CAR-T cell therapy has achieved remarkable success in hematologic malignancies, its application in solid tumors remains significantly more challenging. In this context, gene editing technologies have emerged as a promising strategy to overcome these barriers by reprogramming CAR-T cells (121). Site-specific integration of the CAR cassette into an endogenous gene locus represents an attractive method. For example, by placing CAR expression under the control of the endogenous *TRAC* promoter, Eyquem et al. developed an effective approach to promote memory-like phenotypes and long-term persistence of CAR-T cells, thereby boosting tumor elimination (122). Moreover, non-viral *PDCD1*-integrated anti-CD19 CAR-T cells, produced by CRISPR/Cas9 technology, was demonstrated to achieve high safety and efficacy in treating relapsed/refractory aggressive B-NHL (123). One recent study proposed a strategy to enable simultaneous gene editing at multiple loci, in which the *CAR* gene was knocked into the *TRAC* locus together with cytokine knock-in at *NR4A2* or *RGS16* locus, allowing endogenous regulatory elements to specifically control cytokine expression in the TME (124). In this way, it overcomes the limitation of synthetic promoters that relies on short DNA regulatory fragments and also enables tumor-restricted local delivery of pro-inflammatory cytokines, thus significantly increasing the safety of conventional armored CAR-T cells. In addition to site-specific integration approaches, base editing further expands the scope of gene editing technology in engineering CAR-T cells. Mu et al. designed a method to produce allogeneic universal CAR-T cells by introducing the T316I mutation into the *LCK* locus using a cytosine base editor (CBE) tool, rendering CAR-T cells resistant to the immunosuppressive agent dasatinib, while *TRAC* and *B2M* were also disrupted to avoid graft-versus-host disease (GVHD) and immune rejection (125). Furthermore, prime editing provides a versatile platform for CAR-T cell engineering through the introduction of point mutations and fragment insertion. For instance, prime editing technology has been used to simultaneously disrupt *TRAC*, *B2M* and *PDCD1*, thereby generating universal anti-GD2 CAR-T cells with promising clinical performance (126). Additionally, genome-wide CRISPR/Cas screening has been applied to identify critical genes that negatively regulate T cell function, as well as those essential for T cell survival and activity, thus providing potential gene editing targets for optimizing CAR-T cell therapy in treating solid tumors.

**Figure 3:**
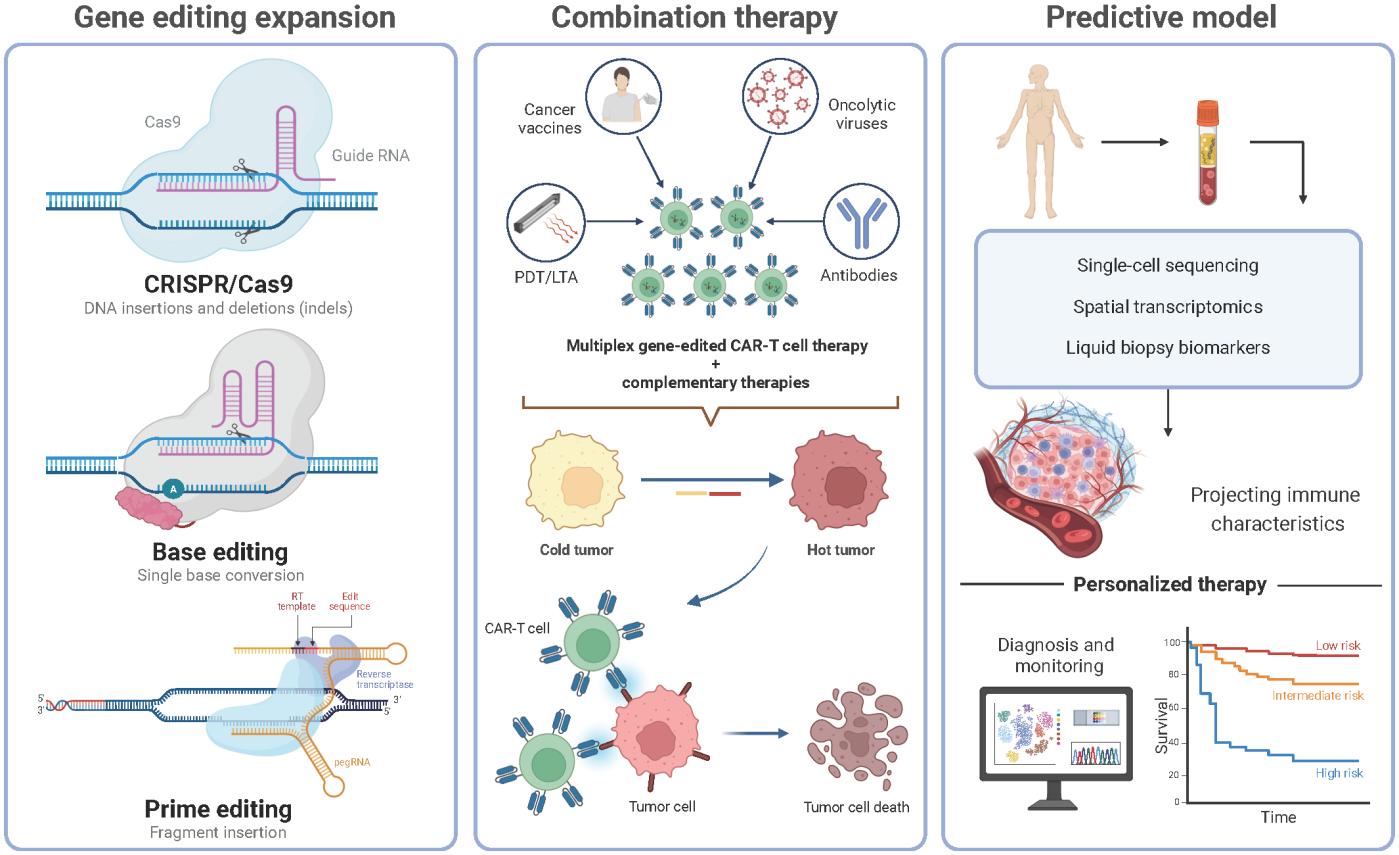
Future perspectives of gene-edited CAR-T cell therapy against solid tumors. Optimizing gene-edited CAR-T cell therapy to overcome the limitations against solid tumors points toward several promising avenues. (1) The development of diverse gene editing technologies enables versatile rewiring of CAR-T cells to enhance antitumor functions. (2) The combination of multiplex gene editing and synergistic therapeutic approaches is driving CAR-T cell therapy toward greater efficacy and safety. (3) The establishment of individualized predictive models is emerging as an important direction for enabling more precise and personalized therapeutic strategies. PDT, photodynamic therapy; LTA, laser thermal ablation. Created with BioRender.com.

Taken together, the continuous development of gene editing technologies helps CAR-T cells to address the complexity of the TME (**Figure 3**). These diverse emerging approaches span a spectrum from precise single-site gene alteration to the construction of elaborate functional circuits. They provide a multilayered and adjustable technical foundation for expanding the boundaries of CAR-T cell therapy and overcoming TME barriers, thereby propelling it toward a more efficient, controllable, and clinically translatable future.

### From multiplex gene editing to combination therapy

The state and immune function of T cells are governed by highly complex and interconnected molecular regulatory networks (127, 128). Hence, single-gene editing is often insufficient to fully reinvigorate CAR-T cell activity in solid tumors. In contrast, multiplex gene editing strategies enable coordinated engineering of CAR-T cells through simultaneous modulation of multiple key genes or addition of functional modules, thereby more significantly boosting CAR-T cell function. For example, CAR-T cells exogenously co-expressing IL-7 and IL-15 can synergistically promote differentiation toward a memory-like phenotype and enhance cell proliferation, accordingly markedly improving the antitumor efficacy (129, 130). CAR-T cells can also be equipped to secrete functional molecules, such as antibodies (e.g., anti-PD-L1 nanobody, CD19-CD3 bispecific T cell engager) and chemokines (e.g., CXCL10, CCL19, and CCL21), which enables local remodeling of the TME and recruitment of bystander immune cells (131–135). In addition, a high-throughput CRISPR functional screening study suggested that a combinatorial knockout strategy (e.g., *RHOG*, *Fas*) can achieve coordinated optimization of transcriptional regulation, metabolic pathways, and signal transduction in CAR-T cells for more effective tumor clearance (136). A dual-engineering design incorporating anti-HER2 CAR and anti-MSLN synNotch circuits was also developed to enable CAR-T cells to specifically express matrix metalloproteinases (MMPs) to degrade dense ECM upon recognizing tumor antigens, thus enhancing CAR-T cell infiltration into solid tumors (137).

Solid tumors frequently exhibit a “cold tumor” phenotype characterized by poor immune cell infiltration and low inflammatory activity (138, 139). A dense fibrotic stroma, abnormal vasculature, and hypoxic and acidic metabolic conditions collectively create multiple barriers to T cell migration, survival, and effector function (44, 140, 141). Although genetic engineering strategies can partially enhance the resistance of CAR-T cells to immunosuppressive signals within the TME, intracellular modifications of CAR-T cells alone are likely insufficient to completely overcome various barriers of the TME. Therefore, strategies combining CAR-T cells with other therapies aiming to convert “cold tumor” into “hot tumor” have emerged as a promising direction for improving CAR-T cell therapy in solid tumors. Oncolytic virus therapy represents an appealing approach. Engineered viruses (such as AdV@Nap gel (142) and T-VEC (143, 144)) selectively replicate within tumor cells and induce lytic cell death while releasing large amounts of damage-associated molecular patterns (DAMPs), including HMGB1, ATP, and exposed calreticulin. These molecules activate dendritic cells and innate immune responses, thereby amplifying adaptive immune activation. In addition to direct oncolysis, these viruses also facilitate CAR-T cell function by enhancing antigen presentation and supplying local inflammatory signals. Moreover, photodynamic therapy (PDT), laser thermal ablation (LTA) and oncolytic peptides (OLPs) can induce local immune activation, improve immune accessibility within the TME, consequently promoting CAR-T cell infiltration and potency in multiple tumor models (145–148). Furthermore, cancer vaccines that strengthen endogenous immune responses and induce antigen spreading have been proved to improve *in vivo* expansion and cytotoxicity of CAR-T cells (138, 149). It is noteworthy that early clinical studies have shown encouraging preliminary results using these combinatorial therapies (149, 150).

Overall, an integrated therapeutic paradigm extending beyond single-strategy cellular engineering represents a promising future direction for CAR-T cell therapy against solid tumors (**Figure 3**). In this scenario, systematic optimization of CAR-T cell function can first be achieved through intracellular engineering strategies, such as multiplex gene editing, followed by combination with complementary therapeutic approaches, including oncolytic viruses and cancer vaccines. Besides reprogramming CAR-T cells by endogenous gene editing, arming CAR-T cells with exogenous functional modules can remodel the physical structure, metabolic conditions, and immune composition of the TME, thus providing effective strategies for solid tumor treatment together with other therapies. With ongoing advances in synthetic biology, gene editing technologies, and local immune-modulatory strategies, such combinatorial frameworks are expected to substantially expand the potential of CAR-T cell therapy in solid tumors.

### Development of predictive models

Beyond the intrinsic biological limitations of solid tumors, substantial heterogeneity in immune responses among patients represents another major factor contributing to variable outcomes of CAR-T cell therapy. Patients undergoing CAR-T cell treatment often have previously received multiple rounds of chemotherapy or are in advanced disease stages with compromised physiological conditions. As a result, the number, subset composition, and functional status of T cells are severely impaired in these individuals, typically characterized by reduced proportions of naïve or central memory T cells and increased expression of exhaustion-associated phenotypes (151, 152). Even in patients with relatively normal baseline T cell counts, elevated expression of immunosuppressive molecules, such as PD-1 and CTLA-4, may limit the *in vivo* expansion and long-term persistence of CAR-T cells (152, 153). Therefore, establishing predictive and stratification models based on patient-specific immune characteristics is essential for enabling more precise and personalized therapeutic strategies that address diverse clinical needs and improve the success rate of CAR-T cell therapy (**Figure 3**).

Multiple studies have demonstrated that integrated assessment of peripheral immune indicators (including T cell subset composition, immune checkpoint expression, and circulating cytokine levels), together with TME characteristics (such as the abundance of Tregs, MDSCs, and TAMs), can facilitate the construction of prognostic models for risk stratification and optimization of strategies before treatment (12, 151, 154, 155). With the development of single-cell and spatial transcriptomics, researchers are now able to track CAR-T cell expansion and differentiation trajectories *in vivo* with high cellular resolution (156–158). These approaches enable the identification of cellular lineages associated with durable responses, continuously refining predictive models (159, 160).

Concurrently, noninvasive monitoring methods generate dynamic biomarkers that not only reflect treatment responses but also inform patient stratification and individualized therapeutic interventions. Circulating tumor DNA (ctDNA) levels can reflect tumor burden and minimal residual disease (MRD) in real time, and rapid clearance of ctDNA following CAR-T cell infusion has been demonstrated to correlate with durable remission (161–163). Noninvasive epigenomic or methylation analyses based on cell-free DNA (cfDNA) have also shown potential predictive value (164, 165). Based on these predictive tools, personalized CAR-T cell therapy can be tailored for solid tumor-earing patients with distinct immune states. For instance, in patients with insufficient T cell repertoire, co-delivery of cytokines such as IL-7 or IL-15 may enhance the expansion of CAR-T cells (166–168). Conversely, in patients with high immune activation or toxicity risk, controllable strategies, such as suicide switches, inhibitory CARs (iCARs), synNotch-based logic gating and drug-inducible or degradable CAR systems, help to improve the safety of CAR-T cell therapy by providing precise spatiotemporal regulation *in vivo* (169–172). For patients with compromised T cell fitness or limited *ex vivo* expansion capacity, *in vivo g*eneration of CAR-T cells has emerged as an attractive strategy, enabling direct reprogramming of T cells within the body and bypassing the need for *ex vivo* cell manufacturing (173–175).

## Conclusions and perspectives

Driven by continuous breakthroughs, gene editing technology, exemplified by CRIPSR/Cas, is rapidly developing toward higher precision, lower off-target effects, larger-fragment targeted integration, and safer *in vivo* delivery. Progressing from basic tool optimization to clinical translation and cross-disciplinary integration, it has already been widely applied in diverse fields, such as clinical therapeutics, agricultural breeding, and ecological governance, and offers substantial potential. Meanwhile, as an innovative cellular immunotherapy, CAR-T cell therapy has achieved remarkable breakthroughs in the treatment of hematological malignancies, providing renewed hope for patients with relapsed or refractory cancer. While CAR-T cell therapy faces multiple challenges in solid tumors, it also holds great promise beyond cancer, including for autoimmune, cardiac, and senescence-associated diseases (176). Optimizing this therapy through gene editing to address the limitations in solid tumors and expand the scope of application thus represents a future direction with tremendous potential. Several promising trends are noteworthy: (1) Comprehensive analysis of complex regulatory systems: Systematic analysis of transcriptional regulation, epigenetic modification, metabolic fitness, and protein-protein interactions will uncover new targets, thereby offering fundamental guidance for designing and optimizing CAR-T cell therapy. (2) Development of diverse gene editing technologies: Gene editing technology is evolving from single-site correction to multiple types of gene modification continuously, enabling versatile rewiring of CAR-T cells. Combined with multiplex gene editing and synergistic therapeutic strategies, CAR-T cell therapy is developing toward greater efficacy and safety. (3) Development of predictive models: The establishment of individualized predictive models is emerging as an important direction. By integrating patients’ clinical characteristics and cellular parameters, personalized treatment strategies can be precisely formulated, significantly improving therapeutic efficacy and safety. In the future, gene editing technology will continue to progress toward higher efficiency and greater precision, thereby overcoming current bottlenecks and further expanding the applications of CAR-T cell therapy, providing revolutionary breakthroughs in treating a broader spectrum of diseases.
